# Deep learning analysis of reflectance confocal microscopy for basal cell carcinoma diagnosis

**DOI:** 10.1016/j.jdin.2026.04.010

**Published:** 2026-04-22

**Authors:** Ross O'Hagan, Alessandra Acciardi, Marianne Tissot Rodríguez, Jaanvi Mehta, Silvana Ciardo, Antonio Alma, Kristina Navrazhina, Nicholas Gulati, Seher Banu Farabi Atak, Francesca Farnetani

**Affiliations:** a The Kimberly and Eric J. Waldman Department of Dermatology, Icahn School of Medicine at Mount Sinai, New York, New York; b Dermatology Unit, Department of Surgical, Medical, Dental and Morphological Sciences Related to Transplant, Oncology and Regenerative Medicine, University of Modena and Reggio Emilia, Modena, Italy

**Keywords:** artificial intelligence, basal cell carcinoma, deep learning, reflectance confocal microscopy

*To the Editor:* Reflectance confocal microscopy (RCM) provides high-resolution noninvasive imaging for basal cell carcinoma (BCC).^1^ Deep learning performs well in noninvasive image analysis,^2^^,^^3^ including RCM-based BCC diagnosis.^4^^,^^5^ Opportunities remain to improve for RCM-based AI diagnostic performance. The aim of this study was to evaluate the diagnostic performance of a bidirectional long short-term memory (BiLSTM) deep learning model applied to *in vivo* RCM images for the detection of BCC.

This retrospective observational study assessed the diagnostic accuracy of a DL pipeline for classifying BCC. The dataset was collected at the University of Modena and Reggio Emilia (Italy), where clinical data and RCM images (Vivastack-3000) were acquired. The study protocol included 230 histopathologically confirmed lesions: 82 BCC and 148 heterogeneous nonBCC controls (including melanocytic nevi, melanomas and seborrheic keratoses). External validation used 2 nonoverlapping, publicly available independent cohorts with no shared patients. The Memorial Sloan Kettering Cancer Center RCM dataset contained 61 lesions with 39 BCC and 22 non-BCC including actinic keratosis, lichen planus-like keratosis and desmoplastic trichoepithelioma as well as the Hames RCM dataset cohort of 54 healthy volunteers. Full cohort characteristics in Supplementary Table I, available via Mendeley at https://data.mendeley.com/datasets/7s34drny3w/1.

Individual RCM frames were processed through frozen ResNet50 backbone to extract 2048-dimensional embeddings, concatenated as ordered sequences and classified by BiLSTM in Python (3.12.9). Model training and validation used a five-fold stratified cross-validation design with lesion-level grouping ensuring all frames from given lesion were confined to single fold with no lesion contributing to both training and validation partitions. Performance was evaluated via the area under the receiver operating characteristic curve (AUC) and a representative saliency map was generated to illustrate model predictions.

Across all folds, the model achieved a mean AUC of 0.984 ± 0.012, with fold-specific values ranging from 0.963 to 1.000. Overall accuracy remained high throughout the validation sets. The aggregated results yielded a sensitivity of 90.2%, specificity of 95.3%, and overall accuracy of 93.5%. External validation on the Memorial Sloan Kettering Cancer Center cohort had AUC of 0.735. Combined external validation of (Memorial Sloan Kettering Cancer Center and Hames cohorts *n* = 115) yielded AUC of 0.864 (Figs 1 and 2).

**Fig 1:**
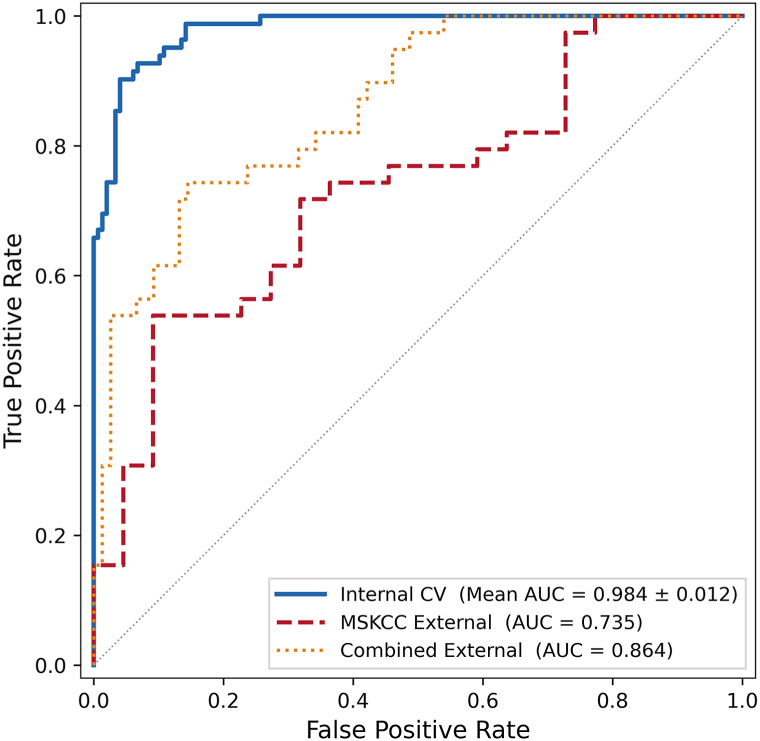
Mean ROC curve of the BiLSTM model across 5-fold cross-validation and external cohort. *BiLSTM*, Bidirectional long short-term memory; *MSKCC*, Memorial Sloan Kettering Cancer Center.

**Fig 2:**
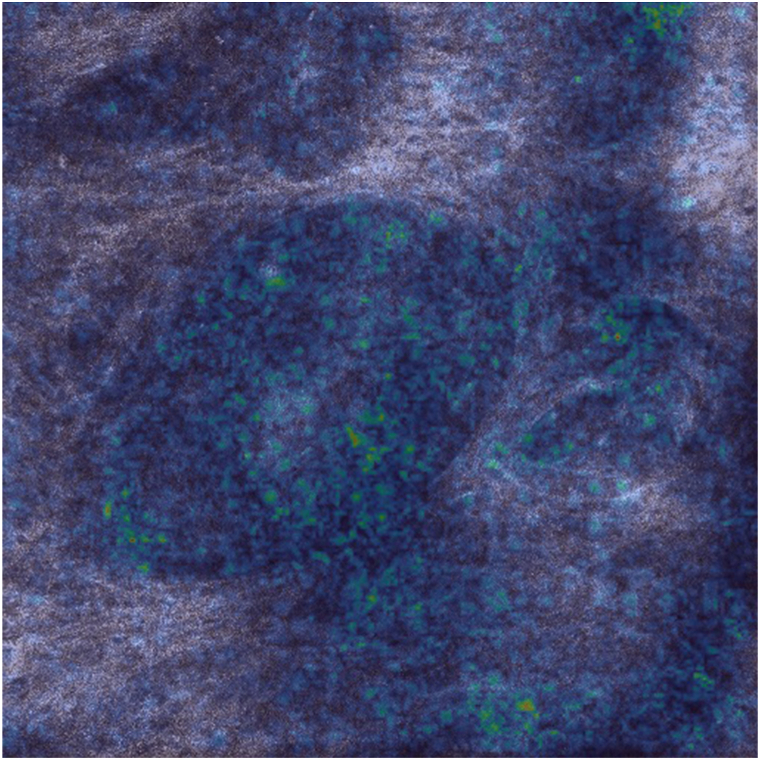
Saliency map features on BCC case. *BCC*, Basal cell carcinoma.

This study demonstrates that a BiLSTM-based deep learning model applied to in vivo RCM images can achieve high diagnostic accuracy in distinguishing BCC from other cutaneous lesions. Unlike prior approaches, our study required no manual annotation^4^ and included histopathology confirmation for all internal training and test cases.^5^ We demonstrate feasibility of external validation using 2 publicly available cohorts. The reduction in AUC from 0.984 to 0.864 reflects domain shift and the broad diagnostic spectrum of the external cohort. A limitation of our study is that these results are specific to the datasets, cases were stratified by lesion not patient, and that our study’s BCC prevalence is not reflective of the general population. While limited by its retrospective design and limited clinically challenging mimickers our study addresses the scarce literature regarding the utility of deep learning for BCC diagnosis via RCM and demonstrates feasibility with multi-institutional external validation.

This study demonstrates the potential of BiLSTM-based deep learning as an alternative approach for RCM analysis. Further prospective studies incorporating additional interpretability analysis and clinically challenging mimickers of BCC are warranted.

### Declaration of generative AI and AI-assisted technologies in the writing process

AI was not used for manuscript composition, except for spelling and grammar checks.

## Conflicts of interest

Dr Gulati is a consultant for the following companies: Almirall, Daiichi Sankyo, and Primus Pharmaceuticals. The rest of the authors declare no conflicts of interest. Drs O'Hagan, Acciardi, Alma, Navrazhina, Atak, Farnetani, and Authors Rodriguez, Mehta, and Ciardo have no conflicts of interest to declare.
